# Caregivers’ Grit Moderates the Relationship Between Children’s Executive Function and Aggression

**DOI:** 10.3389/fpsyg.2020.00636

**Published:** 2020-04-21

**Authors:** Bess Y. H. Lam, Adrian Raine, Annis L. C. Fung, Yu Gao, Tatia M. C. Lee

**Affiliations:** ^1^Department of Rehabilitation Sciences, The Hong Kong Polytechnic University, Kowloon, Hong Kong; ^2^Department of Psychology, University of Pennsylvania, Philadelphia, PA, United States; ^3^Department of Psychiatry, University of Pennsylvania, Philadelphia, PA, United States; ^4^Department of Criminology, University of Pennsylvania, Philadelphia, PA, United States; ^5^Department of Social and Behavioural Sciences, City University of Hong Kong, Kowloon, Hong Kong; ^6^The Graduate Center, The City University of New York, New York, NY, United States; ^7^Brooklyn College, The City University of New York, New York, NY, United States; ^8^Laboratory of Neuropsychology, The University of Hong Kong, Pokfulam, Hong Kong; ^9^Laboratory of Cognitive Affective Neuroscience, The University of Hong Kong, Pokfulam, Hong Kong; ^10^The State Key Laboratory of Brain and Cognitive Sciences, The University of Hong Kong, Pokfulam, Hong Kong; ^11^Institute of Clinical Neuropsychology, The University of Hong Kong, Pokfulam, Hong Kong

**Keywords:** executive function, proactive aggression, reactive aggression, perseverance, grit

## Abstract

**Objective:**

Previous studies have shown that the impairment of executive function is positively related to aggression in children and adolescents. What is worth investigating is the moderator of such a relationship so that aggressive behavior can be reduced effectively in those who have executive function problems. The present study examined the association between executive function and two major subtypes of aggression (proactive and reactive aggression) and whether their caregivers’ grit (perseverance) moderated such relationship.

**Method:**

Executive function and reactive and proactive aggression were assessed in 254 children and adolescents aged 8–19 years old, and their caregivers’ grit was measured.

**Results:**

Results show that caregivers’ grit plays a significant role in moderating the relationship between children’s executive function and proactive aggression after controlling for the covariates including the children’s age, gender, and family income. Specifically, children’s executive function became more negatively associated with proactive aggression when caregivers’ grit was high while the association was positive when it was low. On the other hand, the association between children’s executive function and reactive aggression did not vary across different levels of caregivers’ grit.

**Conclusion:**

These findings suggest that proactive aggression may be reduced in those who have better executive function by enhancing their caregivers’ grit, which inform the design of interventions in adjunct with the current approach (e.g., executive function training) to reduce aggression in children and adolescents in the community.

## Introduction

A large body of research has documented the linkage between deficits in executive function and aggression in children and adolescents (e.g., Morgan and Lilienfeld, 2000; Ogilvie et al., 2011; Poland et al., 2016). As widely accepted, executive functions refer to “the directing cognitive processes that enable purposeful and goal-directed behavior” (Oosterlaan et al., 1998; Anderson, 2002). According to Nigg (2006), executive functions are composed of mental processes ranging from planning and inhibition of inappropriate responses to decision making, which serve to optimize one’s behavior and responses to adapt to the changing environment. Diamond (2013) defined it as a goal-directed cognitive process consisting of three core components, namely, cognitive flexibility (mental flexibility), inhibition (inhibitory control), and working memory. Although the association between executive function and aggression has been established, studies pertaining to the moderators of such a relationship are scarce, and most of them focused on the characteristics of the children and adolescents themselves (e.g., Hoaken et al., 2003). In addition to the contribution by the children and adolescents themselves, would their caregivers play a role in the relationship between executive functions and aggression? The social cognitive theory (Bandura, 1983) suggests that both internal states and environmental factors influence one’s aggression, which is one part of a decision-making process. What remains uncertain is whether the characteristics of caregivers of children (specifically the trait of perseverance) can affect such a relationship. By understanding the underlying mechanism of the relationship between executive function and aggression, we may be able to help reduce aggression in children and adolescents in the community. Therefore, the present study aimed to investigate the moderating role of caregivers’ trait of perseverance in the relationship between executive function and aggression in children and adolescents.

Impaired executive functions are often associated with a range of outcomes including worsening academic performance (Diamond and Lee, 2011), socially inappropriate behavior, aggressiveness, and impulsive behavior, which are all important to the etiology of antisocial behavior (Fuster, 2000; Mesulam, 2002; Moffitt et al., 2011; Holmes et al., 2016). Among all these outcomes of executive functions, aggression which increases the social and economic burden in the community has been widely examined (e.g., Morgan and Lilienfeld, 2000; Ogilvie et al., 2011; Schoemaker et al., 2013). Specifically, the meta-analysis of 126 studies performed by Ogilvie et al. (2011) found that antisocial participants (clinical psychiatric diagnoses, violation of legal or social norms, and aggressive or violent behavior) have worse executive functions compared to controls (effect size = 0.44). One of these studies (Séguin et al., 2004) found that executive function was negatively related to physical aggression. To go beyond the studies on adults, a meta-analysis (Schoemaker et al., 2013) found similar findings in children of preschool age (effect size = 0.22). For instance, Dennis and Brotman (2003) found a significant association between aggression and executive function in children. All of these findings suggested that the inability to inhibit and plan appropriate responses as well as the lack of cognitive flexibility would lead to more aggressive behaviors in children and adolescents.

Yet previous findings related to the relationship between specific subtypes of aggression and impaired executive function were inconsistent across age groups, which warrants investigation. Two prominent forms of aggression consist of reactive and proactive aggression. Reactive aggression is characterized by impulsivity, hostility–aggression, social anxiety, and lack of close friends, while proactive aggression describes instrumental, organized, and “cold-blooded” behaviors (Raine et al., 2006). A number of studies found that the impairment in response inhibition and planning ability (two types of executive functions) was primarily related to reactive aggression but not proactive aggression in children (Ellis et al., 2009; Thomson and Centifanti, 2018). The findings of another study also supported that the deficits in executive function predicted reactive aggression (Giancola et al., 1996). However, other recent studies found that different types of executive ability were differentially associated with reactive and proactive aggression in children and adults (Poland et al., 2016; Hecht and Latzman, 2018). Specifically, proactive aggression increased with higher levels of working memory, while reactive aggression decreased with goal-oriented inhibition and increased with flexibility levels in adults (Hecht and Latzman, 2018). Another study (Poland et al., 2016) found that proactive and reactive physical aggression, as well as proactive relational, were all negatively associated with executive function inhibition, while proactive relational aggression was positively associated with working memory in children. Baker et al. (2019) found similar results regarding reactive and proactive aggression, and they also found that single-parent status increased these two subtypes of aggression in children. Given previous inconsistent findings pertaining to the two subtypes of aggression, this study sought to examine whether deficits in executive functions would increase reactive–proactive aggression in children and adolescents.

With all these findings taken together, different aspects of executive function are differentially related to the two subtypes of aggression. Based on the aforementioned findings, there is a trend suggesting that executive function (inhibition) deficits increase reactive and proactive aggression, whereas working memory is positively related to proactive aggression. Previous inconsistent findings might be due to the fact that some of the previous studies were based on samples of adults using different measurement tools for executive functions. Hence, whether the findings in adults apply to children and adolescents remains questioned (e.g., Hecht and Latzman, 2018). By understanding the relationship between executive function and two subtypes of aggression in children and adolescents, it would be beneficial for developing effective interventions targeting reactive and proactive aggression.

Although more light has been shed on the relationship between executive function and aggressive behaviors in children and adolescents in recent literature (e.g., Schoemaker et al., 2013), by which mechanism executive function leads to aggression is yet to be investigated. In previous literature, it has been long evidenced that caregivers play a significant role in the psychological and cognitive development in children and adolescents (Carneiro and Heckman, 2003; Aunola and Nurmi, 2005; Braza et al., 2015; Liu and Wang, 2015; Foley and Hughes, 2018; Hughes and Devine, 2019). Therefore, it is speculated that caregivers’ characteristics and attitudes (e.g., persevering characteristics when facing difficulties) also have an impact on children who have executive function problems and act aggressively.

Caregivers’ characteristics and attitudes have been suggested to moderate both executive function (e.g., Schroeder and Kelley, 2010; Valcan et al., 2018; Hughes and Devine, 2019) and aggression (e.g., Sulik et al., 2015; Jung et al., 2018) as well as the relationship quality with their children (e.g., Pai and Ha, 2012). These findings suggested that positive caregivers’ factors (e.g., parental warmth and attitudes to the child) protected children and adolescents from executive function deficits and aggressive problems. For instance, Hughes and Devine (2019) suggested that parental influence, negative parent–child interactions, and parenting scaffolding significantly affected children’s executive functions. In another study in children, it was found that non-cognitive skills are the strongest among those who have engaged parents (Carneiro and Heckman, 2003). In addition, it was found that parental emotionally stable personality trait had a positive impact on children’s attention (van Aken et al., 2007). With regard to aggression, Jung et al. (2018) suggested that parental stimulation of development and tolerating parental attitudes might act as a protective factor of externalizing, rule-breaking, and aggressive behaviors in the children who were economically disadvantaged. In contrast, single-parent status, which is an environmental factor, posed a risk for more aggressive behaviors in children and adolescents (e.g., Sulik et al., 2015). Moreover, a high level of psychological control exercised by mothers combined with high affection was associated with increased internalizing and externalizing behavioral problems in children and adolescents (Aunola and Nurmi, 2005). In addition, emotionally stable and conscientious personality trait in caregivers protected the children from being aggressive (van Aken et al., 2007; Oliver et al., 2009). In terms of the relationship quality between caregivers and their children, a differential effect of the widowed parents’ personality traits was found (Pai and Ha, 2012). Specifically, they found that widowed parents with an agreeable personality had more positive interactions, while those with extraversion or openness to experience had more negative interactions with their children. Along the same lines, a study found that infant attachment security was sensitive to both static and dynamic aspects of parenting quality across the first year (Kim et al., 2017). Furthermore, caregivers’ personality was suggested to be the inner resource that moderated the impact on parenting (Kochanska et al., 2007; Koenig et al., 2010). These findings about the relationship quality and caregivers’ personality suggest that children and adolescents may prefer more stable and positive caregivers’ characteristics, which in turn induce a positive impact on children’s executive function and aggression. Taken altogether, caregivers’ characteristics play a significant role in the cognitive and psychological development of children and adolescents. However, previous findings were mainly based on Western samples, while the characteristics of caregivers vary across cultures (Liu et al., 2005; Porter et al., 2005). For instance, Chinese mothers scored higher on overall involvement than Canadian mothers did, while the latter ones scored higher than the former ones on encouragement of autonomy during mother–child interaction (Liu et al., 2005). In addition, whether parental influence moderates the relationship between the relationship between executive function and aggression in children and adolescents requires further investigation. Hence, the present study aimed to explore whether parents’ grit (persevering characteristics in the face of difficulties) would moderate the relationship between executive function and reactive and proactive aggression in Chinese children and adolescents.

As suggested by Seligman and Csikszentmihalyi (2014), prior literature has often focused on studying the pathological side of psychology across age groups including children and adolescents. Positive psychology, which promotes studying the bright side of psychology such as happiness and perseverance, has emerged in recent decades. As there is a growing amount of research studies investigating the positive aspects of psychology, Duckworth et al. (2007) has pioneered research studies pertaining to “grit,” which refers to the perseverance and passion for long-term goals and the persevering characteristics in the face of difficulties and challenges. Subsequent studies showed that grit was associated with a wide range of positive outcomes including success in military selection courses, at high school, and in marriage; good health behaviors; lower incarceration rates; more happiness; and life satisfaction (Heckman and Rubinstein, 2001; Heckman, 2006; Singh and Jha, 2008; Chiteji, 2010; Reed et al., 2013; Eskreis-Winkler et al., 2014). Moreover, grit also was found to be associated with better self-control and lower risky behaviors (Romer et al., 2010; Duckworth and Gross, 2014). For instance, it was suggested that grit acted as a protective factor against substance abuse and other risky behaviors (Guerrero et al., 2016). Since being a caregiver of a child or adolescent who has continuous and drastic changes in terms of psychological, cognitive, and physical aspects over time, it is crucial for caregivers to have persevering characteristics in order to support the positive development of children and adolescents in various aspects. Taken together, the present study aimed to take a more optimistic approach instead of a traditional pathological perspective (e.g., caregivers’ schizotypal personality trait) in studying how caregivers’ characteristics, specifically grit, could help attenuate the relationship between executive function problems and two subtypes of aggression in children and adolescents. In terms of executive functions, the current study specifically examined children and adolescents’ planning, inhibition, processing, and problem-solving skills.

Grit and executive function are two distinct constructs which are supported by previous literature and the finding of authors’ previous study. In terms of definition, executive functions refer to “the directing cognitive processes that enable purposeful and goal-directed behavior” (Oosterlaan et al., 1998; Anderson, 2002). According to Nigg (2006), executive functions are composed of mental processes ranging from planning and inhibition of inappropriate responses to decision making, which serve to optimize one’s behavior and responses to adapt to a changing environment. Diamond (2013) defined it as a goal-directed cognitive process consisting of three core components, namely, cognitive flexibility (mental flexibility), inhibition (inhibitory control), and working memory. On the other hand, grit refers to the perseverance and passion for long-term goals and the persevering characteristics in the face of difficulties and challenges. In a previous study by the authors, a non-significant correlation between grit and executive function was found in children and adolescents aged between 8 and 19 years (*r* = −0.06, *p* = 0.31) (Lam et al., 2017). Specifically, this previous study investigated the effect of omega-3 supplementation on executive functions and grit, but no significant findings were found in relation to trait grit. In addition, the relationship between the two was investigated in adults. For example, the relationship between neurocognitive functions including executive function and trait grit were investigated in the adult participants with and without HIV (Moore et al., 2018). It was found that higher grit was related to better neurocognitive performance in those with HIV, while they were unrelated in those without HIV. Along the same line, high levels of grit were found to be beneficial in solving a problem, which is a proxy of executive function (Mauro et al., 2009; Pierro et al., 2012). With all these results taken together, although executive functioning and trait grit may be related, they are two different constructs, and grit is not a proxy of executive function. Therefore, the present study treated executive function and trait grit as two distinct constructs.

In order to address the literature gaps, the present study attempted to examine the following:

1. the association between executive function and two subtypes of aggression (reactive and proactive aggression), in which it was hypothesized that both reactive and proactive aggression would be negatively related to executive function, and
2. whether caregivers’ grit moderated such a relationship, in which it was hypothesized that more persevering caregivers would help reduce the negative effect of the executive function on reactive and proactive aggression.

## Materials and Methods

### Participants

Promotional letters were sent to invite different primary and secondary schools in the community to join the present study. Among about 2,000 students who were eligible for participation from these schools, a total of 254 schoolchildren and one of their caregivers in Hong Kong were finally recruited and participated in the present study on a voluntary basis. The participation rate was about 12.7%. These schoolchildren [164 (64.6%) male, 90 (35.4%) females] were aged from 8 to 19 years old [mean age = 11.09, standard deviation (SD) = 2.49]. The majority of the caregivers were the biological mothers (*N* = 185, 72.8%) or fathers (*N* = 49, 19.3%), and the remaining ones were either adoptive parents or caregiving relatives. Ethical approval was provided by the Research Committee of the City University of Hong Kong. Caregivers’ and participants’ written informed consent was obtained. Approval was also obtained from all principals, vice principals, and school administrators. Participation was voluntary and anonymous. All procedures performed in studies involving human participants were in accordance with the ethical standards of the institutional and/or national research committee and with the 1964 Helsinki declaration and its later amendments or the American Psychological Association (APA) ethical standards in the treatment of human participants.

Children’s executive function was measured individually in the laboratory. Reactive and proactive aggression in the children and adolescents and their caregivers’ grit as well as their demographic information (e.g., age, gender, family income, and relationship with the children) were measured by self-report scales. All measures were translated and back-translated to Chinese and administered by trained research assistants. Upon completion of the study, the participants received monetary compensation and debriefing about the study purposes.

### Measures

#### Youth’s Executive Function

Executive function, which encompasses planning, processing, and problem-solving skills, was assessed by the Tower of London (TOL) test (Shallice, 1982). In this task, participants were asked to rearrange a set of three beads that were placed on three rods with descending heights strategically from a preset starting position. Specifically, the participants were given a different set of beads and rods to match the original set of beads. Based on the participants’ performances in this task, three subscores of TOL were recorded: the accuracy of the solution, which referred to the rate of correct moves (TOL accuracy); the efficiency of the solution, which referred to the total time consumed to solve all solutions (TOL time); and the number of moves performed to rearrange the beads (TOL move). TOL is sensitive to problem difficulty as supported by previous studies (Baker et al., 1996). The executive function composite score was calculated by summing up three standardized subscores of TOL with the score reversion of TOL time and TOL move. The higher the TOL composite score, the better the executive function of the participants. The computation of the TOL composite score was adopted from a previous study (Lam et al., 2014). The psychometric construct is similar in adults (age range = 18–32 years) (Kaller et al., 2012) and in children and adolescents (age range = 7–15 years) (Culbertson and Zillmer, 1998).

#### Youth’s Proactive and Reactive Aggression

Reactive and proactive aggression was assessed using the Reactive–Proactive Aggression Questionnaire (RPQ) (Raine et al., 2006). The RPQ is a self-report scale containing 23 behavioral items. Sample items assessing reactive aggression included “Got angry or mad or hit others when teased,” “Reacted angrily when provoked by others,” and “Felt better after hitting or yelling at someone,” while those assessing proactive aggression included “Had fights with others to show who was on top,” “Vandalized something for fun,” and “Used physical force to get others to do what you want.” Participants were asked to rate these on a 3-point scale (0 = “never,” 1 = “sometimes,” and 2 = “often”). The total RPQ score was also computed by the summation of reactive and proactive scores. Internal consistency in Chinese schoolchildren aged 11–15 years has previously been reported as 0.89 for proactive aggression, 0.88 for reactive aggression, and 0.83 for general aggression (Fung et al., 2009). In this study, internal consistency reliability was 0.86 (total aggression), 0.83 (proactive aggression), and 0.81 (reactive aggression).

#### Caregivers’ Grit

Caregivers’ grit was assessed by the eight-item Grit-S (Duckworth and Quinn, 2009). Participants were asked to rate themselves on a 5-point Likert scale from 1 (not like me at all) to 5 (very much like me). For instance, participants rated items such as “Setbacks don’t discourage me” and “I finish whatever I begin.” This scale has received reasonable internal consistency (0.73–0.83) in previous studies (Duckworth and Quinn, 2009). In the current sample, Cronbach’s alpha was 0.57.

### Statistical Analysis

Correlations were used to assess the association of demographic information with children’s aggression and executive functions, and caregivers’ grit (Table 1). To examine the moderation of caregivers’ grit on the relationship between children’s executive function and aggression, moderation analyses were performed using the SPSS PROCESS macro (Hayes, 2013). The SPSS PROCESS macro is a computational tool for path analysis-based moderation and mediation analysis as well as their combination as a “conditional process model.” Children’s executive function was the independent variable (IV); caregivers’ grit was the moderator (M); and reactive/proactive aggression was the dependent variable (DV). A *p*-value less than or equal to 0.05 regarding the executive function × caregivers’ grit (IV × M) interaction indicates significant moderation. Specifically, this significant moderation would indicate that the effect of executive function on reactive/proactive aggression varied across the level of caregivers’ grit. The moderated effects of executive function on reactive/proactive aggression at five levels of caregivers’ grit (10th, 25th, 50th, 75th, and 90th percentiles) were also analyzed. In the moderation analyses of the present study, the children’s age, gender, and family income, which were found to be related to executive function and aggression in the present study and prior studies (Keenan and Shaw, 1997; Carlson, 2005; Raaijmakers et al., 2008; Hughes et al., 2009; Braza et al., 2015), were included as covariates. In addition, proactive or reactive aggression was controlled for in order to delineate the specific effects for each subtype of aggression.

**TABLE 1:** Intercorrelations between study variables (P’s and C’s self-report ratings^a^).

	**1**	**2**	**3**	**4**	**5**	**6**	**7**
1. C-age	–	–	–	–	–	–	–
2. P-income^b^	−0.14*	–	–	–	–	–	–
3. P-grit	–0.004	0.15*	–	–	–	–	–
4. C-executive function^c^	0.18**	–0.09	–0.11	–	–	–	–
5. C- General aggression	–0.05	–0.01	0.09	−0.14*(−0.05,−0.22**)^d^	–	–	–
6. C-reactive aggression	−0.14*	–0.06	0.07	−0.11(−0.08,−0.12)^d^	0.91***	–	–
7. C-proactive aggression	0.12	0.07	0.10	−0.14*(0.02,−0.26**)^d^	0.79***	0.48***	–
Mean (SD)	11.09 (2.49)	4.91 (2.52)	23.11 (4.04)	0.09 (2.46)	6.46 (5.22)	5.18 (3.61)	1.28 (2.41)

* *p* ≤ 0.05, ** *p* ≤ 0.01, *** *p* ≤ 0.001. ^*a*^P = Caregivers’ self-report, C = Children’s self-report. ^*b*^1 = HK$5,000 or below, 2 = HK$5,001–10,000, 3 = HK$10,001–15,000, 4 = HK$15,001–20,000, 5 = HK$20,001–25,000, 6 = HK$25,001–30,000, 7 = HK$30,001–35,000, 8 = HK$35,001–40,000, 9 = HK$40,001–45,000, 10 = HK$45,001–50,000, 11 = HK$50,001 or above. ^*c*^Standardized score. ^*d*^(Correlation estimates when caregivers’ grit is low, correlation estimates when caregivers’ grit is high); high-level grit and low-level grit are computed by a median split of caregivers’ grit scores.

## Results

The mean and SD of each study variable and covariate are stated in Table 1.

### Associations Between Major Study Variables and Demographic Information

The children’s age was significantly related to the composite standardized score of the TOL test (*r* = 0.18, *p* < 0.01) and reactive aggression (*r* = −0.14, *p* < 0.05), while children’s gender was significantly associated with the total RPQ score [*t*(252) = −2.58, *p* = 0.01] and proactive aggression score [*t*(252) = −3.91, *p* < 0.0001]. In addition, family income was significantly related to caregivers’ grit (*r* = 0.15, *p* < 0.05). All other associations with demographic information were not significant. Children’s executive functions were negatively related to general (*r* = −0.14, *p* = 0.02), proactive (*r* = −0.14, *p* = 0.02), and reactive aggression (*r* = −0.10, *p* = 0.08) as well as caregivers’ trait of perseverance (*r* = −0.14, *p* = 0.02). Caregivers’ trait of perseverance was not significantly related to general, proactive, and reactive aggression (*p* > 0.05). In terms of the correlation between children’s executive function and general aggression, they were negatively related to each other in those with a high level of caregivers’ grit (*r* = −0.22, *p* = 0.01) but not in those with a low grit level (*p* > 0.05). Similar results were found for the relationship between children’s executive function and proactive aggression in those with a high level of caregivers’ grit (*r* = −0.26, *p* = 0.002) but not in those with a low grit level (*p* > 0.05). Such correlations were not significant for reactive aggression in both levels of caregivers’ grit (*p* > 0.05).

### Moderation Analyses

#### Reactive Aggression as the DV

**FIGURE 1:**
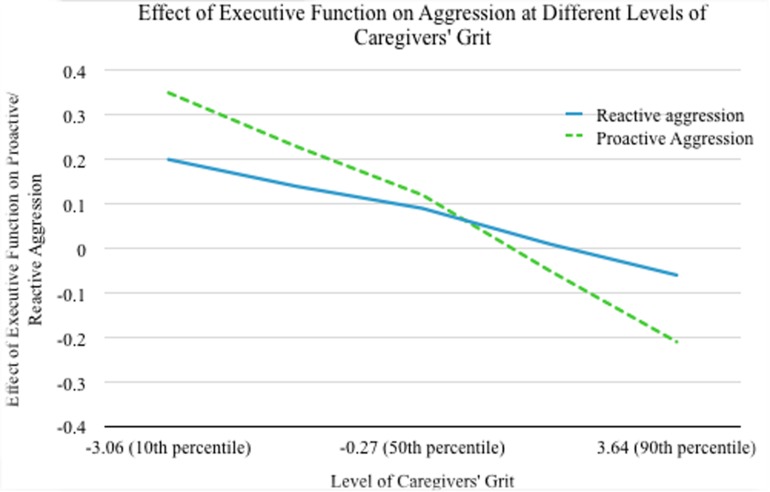
The moderating effects of executive function on reactive/proactive aggression at five levels of caregivers’ grit (10th, 25th, 50th, 75th, and 90th percentiles) were examined. The results showed that the moderating effects of executive function on proactive aggression at five levels of caregivers’ grit were significant (*p* < 0.05) except at the 75th percentile of caregivers’ grit (*p* > 0.05) (dotted line). The moderating effects at five levels of caregivers’ grit were not significant for reactive aggression (*p* > 0.05) (solid line).

After controlling for all covariates including the children’s age, gender, family income, and proactive aggression, the coefficients of children’s executive function and caregiver’s grit were not significant (*p* > 0.05), while the IV × M interaction effect was not significant in predicting reactive aggression (*p* > 0.05) (Table 2). Furthermore, the moderated effects of executive function on reactive aggression at five levels of caregivers’ grit (10th, 25th, 50th, 75th, and 90th percentiles) were not significant (*p* > 0.05) (Table 3 and Figure 1).

**TABLE 2:** Moderation results after controlling for all the covariates in the present study.

	**Controlling for the children’s age and gender, family income, and reactive/proactive aggression**		
	
	***B***	***t* (SE)**	***P***
**DV: Children’s reactive aggression**			
IV: Children’s reactive aggression	–0.02	−0.74(0.02)	0.46
Moderator: Caregivers’ grit	0.08	1.27(0.06)	0.21
IV × moderator interaction	–0.04	−1.60(0.02)	0.11
**DV: Children’s proactive aggression**			
IV: Children’s proactive aggression	–0.05	−2.13(0.02)	0.03*
Moderator: Caregivers’ grit	0.10	1.75(0.05)	0.08
IV × moderator interaction	–0.08	−3.86(0.02)	0.0001***

* *p* ≤ 0.05, *** *p* ≤ 0.001.

**TABLE 3:** The moderated effect of executive function on reactive/proactive aggression at five levels of caregivers’ grit (10th, 25th, 50th, 75th, and 90th percentiles).

**Caregivers’ grit^a^**	***B*^b^**	***t* (SE)**	***p***
**DV: Reactive aggression**			
−3.06 (10th percentile)	0.20	1.92 (0.10)	0.06
−1.63 (25th percentile)	0.14	1.83 (0.08)	0.07
−0.27 (50th percentile)	0.09	1.41 (0.06)	0.16
1.68 (75th percentile)	0.01	0.17 (0.07)	0.86
3.64 (90th percentile)	–0.06	−0.64(0.10)	0.52
**DV: Proactive aggression**			
−3.06 (10th percentile)	0.35	3.85 (0.09)	0.0001***
−1.63 (25th percentile)	0.23	3.38 (0.70)	0.0008***
−0.27 (50th percentile)	0.12	2.12 (0.06)	0.03*
1.68 (75th percentile)	–0.05	−0.74(0.06)	0.46
3.64 (90th percentile)	–0.21	−2.34(0.09)	0.02*

* *p* ≤ 0.05, *** *p* ≤ 0.001. ^*a*^Standardized scores. ^*b*^Coefficient of the moderated effect.

#### Proactive Aggression as the DV

After controlling for all covariates including the children’s age, gender, family income, and reactive aggression, the coefficients of children’s executive functions (*B* = −0.05, *t* = −2.13, *p* = 0.03) and the IV × M interaction effect (*B* = −0.08, *t* = −3.86, *p* = 0.0001) were significant in predicting proactive aggression, while the coefficient of caregiver’s grit was not significant (*p* > 0.05) (Table 2). Furthermore, the moderated effects of executive function on proactive aggression at five levels of caregivers’ grit (10th, 25th, 50th, 75th, and 90th percentiles) were analyzed and presented in Table 3 and Figure 1. All moderated effects were significant (*p* < 0.05) except at the 75th percentile of caregivers’ trait of perseverance (*p* > 0.05). Specifically, the association between children’s executive function and proactive aggression became more negative (from *B* = 0.35, *p* = 0.0001 to *B* = −0.21, *p* = 0.02) as the levels of caregivers’ grit increased from *z* = −3.6 to *z* = 3.64.

## Discussion

Executive function is consistently found to be related to aggression in children and adolescents (Morgan and Lilienfeld, 2000; Ogilvie et al., 2011). Multiple moderators of such a relationship including age and gender have been investigated (Keenan and Shaw, 1997; Carlson, 2005; Raaijmakers et al., 2008; Hughes et al., 2009; Braza et al., 2015). Yet prior studies have focused on studying the characteristics of the children and adolescents themselves, largely overlooking the influence of caregivers’ characteristics on such a relationship. The present study examines the association between executive function (planning, inhibition, processing, and problem-solving skills) and two subtypes of aggression (reactive and proactive aggression) and whether caregivers’ grit moderates such a relationship. Overall, the findings of the present study support the major hypothesis of the present study and are consistent with Bandura’s (1983) suppositions with regard to internal states and environmental factors as equal contributors to aggressive behaviors. Specifically, it is found that caregivers’ grit plays a significant role in moderating the relationship between children’s executive function and proactive aggression, regardless of the children’s age, gender, and family income. This finding suggests that in children and adolescents with more persevering caregivers, better executive function (specifically inhibition) leads to reduced proactive aggression, which is consistent with prior findings (Poland et al., 2016; Baker et al., 2019). In contrast, better executive function leads to more proactive aggression in those with less persevering caregivers. By identifying caregivers’ grit as the moderator of the executive function–aggression relationship, it would be more effective in developing treatment components in adjunct with the current approach (executive function training) to target different subtypes of aggression in children who have executive function problems.

### Moderation Effect on Executive Function–Proactive Aggression

The major finding of the present study is that caregivers’ grit moderates the relationship between children’s executive function and proactive aggression while controlling for children’s age, gender, and family income. This is consistent with Bandura’s (1983) suppositions that it is likely that the aggression is contributed by both internal (children’s executive function) and environmental factors (caregivers’ trait of perseverance). Specifically, in children and adolescents with more persevering caregivers, a more negative association between executive function and aggression, specifically proactive aggression, is found. In contrast, better executive function leads to more proactive aggression in those with less persevering caregivers. This finding suggests that caregivers who persevere when facing difficulties and challenges related to child rearing might provide a positive environment for the children. As such, more persevering caregivers might protect the children and adolescents who have better executive functions from being proactively aggressive. This is consistent with prior findings that the way caregivers act and interact with their children and adolescents plays a significant role in the cognitive and psychological development of the latter (Aunola and Nurmi, 2005; Braza et al., 2015).

However, the moderation effect of children’s executive function and caregivers’ grit is not significant for reactive aggression after controlling for all covariates. This differential finding might be because, by definition, proactive aggression, which is defined as the goal-oriented, cold-blooded, and planned subtype of aggression, is more related to executive function and planning when compared to reactive aggression (Poland et al., 2016; Baker et al., 2019). This might explain why the moderation results for these two subtypes of aggression are different after controlling for all these covariates with the present data. Nevertheless, these moderation results show that caregivers’ grit plays a significant role in the relationship between children’s executive function and proactive aggression.

### Differential Association of Executive Function With Reactive and Proactive Aggression

The first hypothesis is not supported in the present study. The results show that executive function is negatively related to aggression, specifically proactive aggression, while the association is not significant for reactive aggression. The non-significant association between executive function and reactive aggression, which is inconsistent with prior literature and the current hypothesis, might be because of different operationalizations of executive functions and aggression across studies (Morgan and Lilienfeld, 2000; Ellis et al., 2009). Specifically, the TOL test is used in the present study, which taps more on the planning, processing, and problem-solving skills. These aspects might be more related to proactive aggression, which is a goal-oriented, and planned aggression, when compared with reactive aggression, which is an impulsive and provoked type of aggression. On the contrary, the study by Ellis et al. (2009) adopted five neuropsychological tests to assess participants’ executive function and two different self-report reactive aggression scales to measure aggression. Besides the variations in the choice of measurement tool, the age groups of the sampled participants differed across studies. For instance, the age group examined in Ellis et al. (2009) and Giancola et al. (1996) was 9–12 and 10–12 years, respectively, whereas the age of the participants in the present study ranged from 8 to 19 years. Last but not least, one more factor that might explain such inconsistent finding is the cultural factor. Specifically, the types and effects of parenting styles vary across cultures (Liu et al., 2005; Porter et al., 2005). For instance, Chinese mothers scored higher on overall involvement than Canadian mothers did, while the latter ones scored higher than the former ones on encouragement of autonomy during mother–child interaction (Liu et al., 2005). The cultural specificity of caregiver–child interactions might also affect the present findings.

### Limitations and Future Directions

The present study suffered from a number of limitations. First, the small size and representativeness of the sample could be improved in future studies. Specifically, the current sample involved children and adolescents from two schools in Hong Kong, which might affect the generalizability of the findings. In fact, the effects of and types of parenting styles are found to vary across cultures (Liu et al., 2005; Porter et al., 2005). For instance, child activity level was related to more authoritative and less authoritarian parenting styles in Chinese but not in US samples. Future studies should address this limitation. Moreover, the cross-sectional nature of this study precludes strong inferences regarding the direction of effects. The pattern of covariation between variables is interpreted to mean that parental characteristics influence children’s aggression. However, it is possible that high levels of disruptive children’s behaviors also influence parental characteristics. The present findings set a foundation for future longitudinal studies to test the direction of effects. Furthermore, the wide age range of the participants might be a confounding factor in the present study. Although age has been controlled for in this study, a narrower age range should be investigated in future studies. Last but not least, the eight-item grit scale did not receive excellent internal reliability in the present data, which might have affected the moderation analyses, particularly when reactive aggression was treated as the DV. Future studies might consider using the full version of the grit scale for more stable internal reliability.

### Implications

The findings of the present study suggest that a higher level of caregivers’ grit might protect children and adolescents who have better executive functions from being proactively aggressive regardless of their age, gender, and family income. These findings will be helpful in building up interventions in relation to aggression in children and adolescents in the community by targeting their caregivers’ grit. For instance, Duckworth (2016) who is the scholar who coined “grit” suggests a number of ways to enhance one’s perseverance including the following: (1) pursue what interests you; (2) practice, practice, practice because we love doing things we are good at; (3) find the purpose of what you do; and (4) have hope that you are going to make it happen. It is noteworthy to take socioeconomic status (SES) into consideration when designing grit interventions for different participants because SES might affect one’s grit development. Alternatively, resilience skills intervention can be conducted to improve caregivers’ perseverance (Kent et al., 2015). Specifically, this intervention was found to be effective in promoting positive emotion, enhancing neurocognitive capacities, and reducing symptoms in veterans with post-traumatic stress disorder (PTSD). By enhancing caregivers’ grit, which in turn reduces aggression in children and adolescents, we can further reduce the social and economic burden on society.

## Data Availability Statement

The datasets generated for this study are available on request to the corresponding author.

## Ethics Statement

The studies involving human participants were reviewed and approved by the Research Committee of the City University of Hong Kong. Written informed consent to participate in this study was provided by the participants’ legal guardian/next of kin.

## Author Contributions

AR, AF, YG, and TL conceived of the study, participated in its design and coordination, and commented on the draft of the manuscript. BL performed the statistical analysis and helped to draft the manuscript. All authors read and approved the final manuscript.

## Conflict of Interest

The authors declare that the research was conducted in the absence of any commercial or financial relationships that could be construed as a potential conflict of interest.
